# Radiosensitizing effect of ellagic acid on growth of Hepatocellular carcinoma cells: an *in vitro* study

**DOI:** 10.1038/s41598-017-14211-4

**Published:** 2017-10-25

**Authors:** Ujjal Das, Sushobhan Biswas, Sreya Chattopadhyay, Anindita Chakraborty, Rakhi Dey Sharma, Asoke Banerji, Sanjit Dey

**Affiliations:** 1 Department of Physiology, Centre for Nanoscience and Nanotechnology, University of Calcutta, 92 APC Road, Kolkata, 700 009 West Bengal India; 2 Division Radiation Biology, UGC-DAE CSR Center Kolkata, Bidhan Nagar, Kolkata, 700 098 West Bengal India; 3 Department of Food & Nutrition, Barrackpore Rastraguru Surendranath College, 85 Middle Road, Kolkata, 700 120 West Bengal India; 4 School of Biotechnology, Amrita Vishwa Vidyapeetham, Amritapuri,Clappana P.O. Kollam District-690 525, Kerala, India; 5 Centre with Potential for Excellence in Particular Area (CPEPA), University of Calcutta, 92 APC Road, Kolkata, 700 009 West Bengal India

## Abstract

Failure of treatment for cancer in clinic by radio/chemotherapy is generally attributed to tumour resistance. Therefore, it is important to develop strategies to increase the cytotoxicity of tumour cells by radiation in combination with unique tumour selective cytotoxic agents. We evaluated the potential of ellagic acid (EA) as an enhancer of oxidative stress in cancer cells. HepG2 cells were treated with EA (10 µM) for 12 h prior to exposure of single 7.5 Gy dose of irradiation. Treatment of HepG2 cells with EA and gamma radiation showed increased reactive oxygen species generation, up regulation of p53 protein expression, decreased survival markers level like p-Akt, p-NF-kB and p-STAT3 which were significantly higher after radiation treatment alone. We also found that combination treatment increased G2/M phase cell population, decreased IL-6, COX–2 and TNF-α expression and caused a loss in mitochondrial membrane potential with decreased level of angiogenesis marker MMP-9. Over expression of Bax and activation of caspase 3 indicated the apoptosis of the cells. The results provided a strong unique strategy to kill cancer cells HepG2, using less radiation dose along with effective pro-oxidant dose of EA.

## Introduction

Hepatocellular carcinoma (HCC) is the most common primary liver malignancy and the sixth most common cancer worldwide^1^. It has an aggressive malignancy with a poor prognosis and is currently the second most common cause of cancer-related mortality. Although more than 80% of the estimated 782,000 new cases of HCC in 2012 occurred in less developed regions of the world. Its incidence is increasing worldwide, including in more developed countries^1^. Many liver cancers are very resistant to radio-and chemo-therapeutic treatment. In view of the fact that radiotherapy fails in the later stages of cancer due to the development of radioresistant property in tumour cells, it is most important in radiobiology to increase the oxidative damage of the tumour cells by using a tumour selective cytotoxic agent. The increase in radiosensitivity is important both for optimizing the radiation dose for tumours and for designing strategies to improve the therapeutic ratio^2^. Understanding of chemopreventive mechanisms of naturally occurring compounds is a key to the future application of such agents for human health.

Ionizing radiation and certain cytotoxic drugs are known to induce oxidative stress in cancer cells through generation of reactive oxygen species (ROS) resulting in imbalance of the redox homeostasis in the cells which is suggested to culminate in cell death^3,4^. Intracellular generation and accumulation of ROS such as superoxide anion, hydrogen peroxide, singlet oxygen, hydroxyl radical and peroxyl radical in the stressed cells overcome the natural antioxidant defence and causing damage to biological macromolecules including nucleic acids, proteins and lipids^5^. The polyphenolic compounds and flavonoids act as an antioxidant in protecting cells from oxidative stress^6–9^. It is reported that polyphenols isolated from blueberries protected red blood cells from ROS *in vitro* as well as *in vivo* ^10^. Flavonoids and polyphenols may also exhibit prooxidant activity, which contributes to therapeutic functions attributed to flavonoids. These compounds could behave as both prooxidants and antioxidants depending on concentration and free radical source. Flavonoids auto-oxidise in aqueous medium and may form highly reactive OH radicals in the presence of transition metals. Moreover, polyphenols may act as substrate for peroxidase and other metalloenzymes, yielding quinone or quinomethide type prooxidant^11–13^. Earlier report had linked polyphenols to ROS production, especially hydrogen peroxide and subsequent apoptosis^14^.

Ellagic acid (EA) is a polyphenol that has been reported to show antiproliferative activity. It causes cell cycle arrest and to induce apoptosis in many human cancer cell lines such as bladder T24, cervical carcinoma (CaSki), leukaemia MOLT-4, breast MCF-7 and Hs 578 T, and prostate DU 145 cells^15–18^. The occurrence of free EA in dietary foodstuffs is rather uncommon. Earlier EA was isolated from seabuckthrone^19,20^. Ellagic acid is usually conjugated with a glycoside moiety (glucose, arabinose, xilose, etc.) or, even more commonly, forms part of polymeric molecules called ellagitannins. Aspirin, sodium salicylate and several other NSAIDs have been shown to inhibit the activation of NF-κB and at higher concentration potentiate the cytotoxic effects of ionizing radiation. Indomethacin inhibits ionizing radiation-induced activation of NF-κB and sensitizes HeLa cells to ionizing radiation- induced cytotoxicity at similar concentration^21–24^. It is also known that curcumin, a polyphenol conferred radiosensitizing effects in prostate cancer cell line by inhibiting the growth of human prostate PC-3 cancer cells and downregulates radiation-induced prosurvival factors^25^. Tanaka *et al*. showed that EA exhibited cancer inhibitory properties in animal liver model. There is no report present till date, showing the radiosensitization effect of EA on any liver cancer cell. Therefore, it is the first report showing radiosensitization effect of EA on hepatocellular carcinoma cell, HepG2. However, it is also important to study the radiosensitizing effect of EA on other hepatocellular carcinoma cells. The overall strategy of this current research is to minimize the radiation dose yet it kills the hepatocellular carcinoma cells with the help of the prooxidant effects of EA.

This study evaluates the potential of ellagic acid (EA) as an enhancer of radiation-induced apoptosis of cancer cells. HepG2 cells treated with EA and gamma radiation showed increased reactive oxygen generation. Excess reactive oxygen species (ROS) damaged the DNA, initiated apoptotic and inflammatory events. The phosphatidyl serine (PS) externalization and loss of mitochondrial membrane potential (MMP) indicated the initiation of apoptosis. The ROS induced DNA damage up regulated tumor protein p53 (p53) translocation to nucleus followed by p21 expression and cell cycle arrest. Combination of IR and EA decreased the level of activated survival markers like p-Akt, phospho-nuclear factor kappa B (p-NF-kB) and phospho-Signal transducer and activator of transcription 3 (p-STAT3). Loss of MMP initiated intrinsic apoptotic pathway. Therefore, the level of active caspase 3 and ratio of pro-apoptotic protein Bax and anti apoptotic protein BCL2 were measured. Combination treatment increased the Bax/BCL2 ratio and active caspase 3 level thereby, induced cellular apoptosis.

## Materials and Methods

### Materials

EA, TBA, DTNB, HEPES were purchased from Sigma Chemical Co. (St. Louis, MO, USA). Antibodies against BCL2, Bax, p53, p21, Matrix metallopeptidase-9 (MMP-9), and anti-rabbit IgG fluorescein isothiocyanate (FITC) and Rhodamine 1,2,3 conjugated secondary antibodies, and 4′,6-diamidino-2-phenylindole (DAPI) were obtained from Santa Cruz (Santa Cruz, CA, USA). 2′,7′-Dichlorofluoroscein diacetate (H_2_DCF-DA) was purchased from Calbiochem of Merck-Millipore (Billerica, MA, United States). Antibody against p-NF-κB, p-STAT3, cyclooxygenase-2 (COX-2) and p-Akt were purchased from Cell Signaling (Beverly, MA, USA). Antibody to detect the level of active caspase 3, annexin-FITC-PI, and JC1 dye were purchased from Becton Dickinson-Biosciences (San Jose, CA, USA). All the cell culture reagents were purchased from Gibco (Waltham, MA, US) and all other reagents used for this study were of highest quality grade.

### EA and cell lines

Ellagic acid (EA) was purchased from Sigma Chemical Co. (St. Louis, MO, USA). Stock solution of EA (1 mM) was prepared in dimethyl sulfoxide (DMSO), and was filter sterilized before use. The human hepatocellular carcinoma cell line HepG2 was purchased by Dr Partha Chakraborty from ATCC (ATCC, CRL-11997). We got the cell line as a kind gift from Dr Partha Chakraborty. The cells were maintained according to the guideline of ATCC. HepG2 cells were cultured in Dulbecco’s Modified Eagle’s high glucose containing medium (DMEM), supplemented with 10% fetal bovine serum (FBS) and 1% penicillin streptomycin solution in an atmosphere of 95% air and 5% CO_2_ in a 37 °C humidified incubator.

### MTT assay

We considered 3-(4, 5-dimethylthiazolyl-2)-2, 5-diphenyltetrazolium bromide (MTT) assay as cell proliferation assay. When viability increases with respect to control for a particular experimental condition, it indicates higher cell proliferation. Therefore, the viable count in MTT assay depicted the cell proliferation status also. The assay was performed independently 3 times (technical replicate n = 3) and each time 6 replicates for one condition was taken (biological replicate n = 6). 10^4^ cells were plated in 96-well plate and incubated overnight for cell seeding. Cells were treated with different concentrations of EA (10, 20, 30, 40, 50, 70, 90, 100 and 200 µM) and different doses of radiation (5, 7.5, 10, 15 Gy) under serum starved condition. After 72 h of incubation, 10 µl of MTT (5 mg/ml) was added to each well. Cells were incubated for 3 h at 37 °C in 5% CO_2_ pressure. The medium was discarded, and the purple formazan crystals were adequately dissolved with 100 µl of DMSO for 10 min on a rocker shaker. The absorbance was measured at 570 nm using an ELISA reader^26^. The cell viability was calculated according to: OD sample/OD control × 100%. After determining the LD_50_ value of HepG2 cells for radiation and EA, a combination treatment was performed. The viability of radiation + EA treated cells was also determined by applying same protocol.

### Experimental Design

After selecting the dose of irradiation, EA and combination, the cells were divided into four groups. After 12 h of treatment with EA (10^−5^ M), the cells were exposed to 7.5 Gy of gamma-irradiation (^60^Co) at the dose rate of 1 Gy/min. Soon after irradiation, the samples were kept in an atmosphere of 95% air and 5% CO_2_ in a 37 °C humidified incubator and harvested at different time points to check the alterations in redox sensitive pathways.

A viable count was carried out by the standard trypan blue exclusion test, to estimate the number of viable cells in each of the following experimental groups.

- Control group: HepG2 cells were treated with 0.1% DMSO, the vehicle.
- Irradiation group: HepG2 cells were treated with 0.1% DMSO vehicle. After 12 h of vehicle treatment, the cells were exposed to 7.5 Gy dose of irradiation.
- EA (10^−5^ M)-treated group: HepG2 cells were treated with 10^−5^ M of EA.
- EA (10^−5^ M) + Irradiation group: HepG2 cells were treated with 10^−5^ M of EA for 12 h followed by 7.5 Gy irradiation.

**Figure 1:**
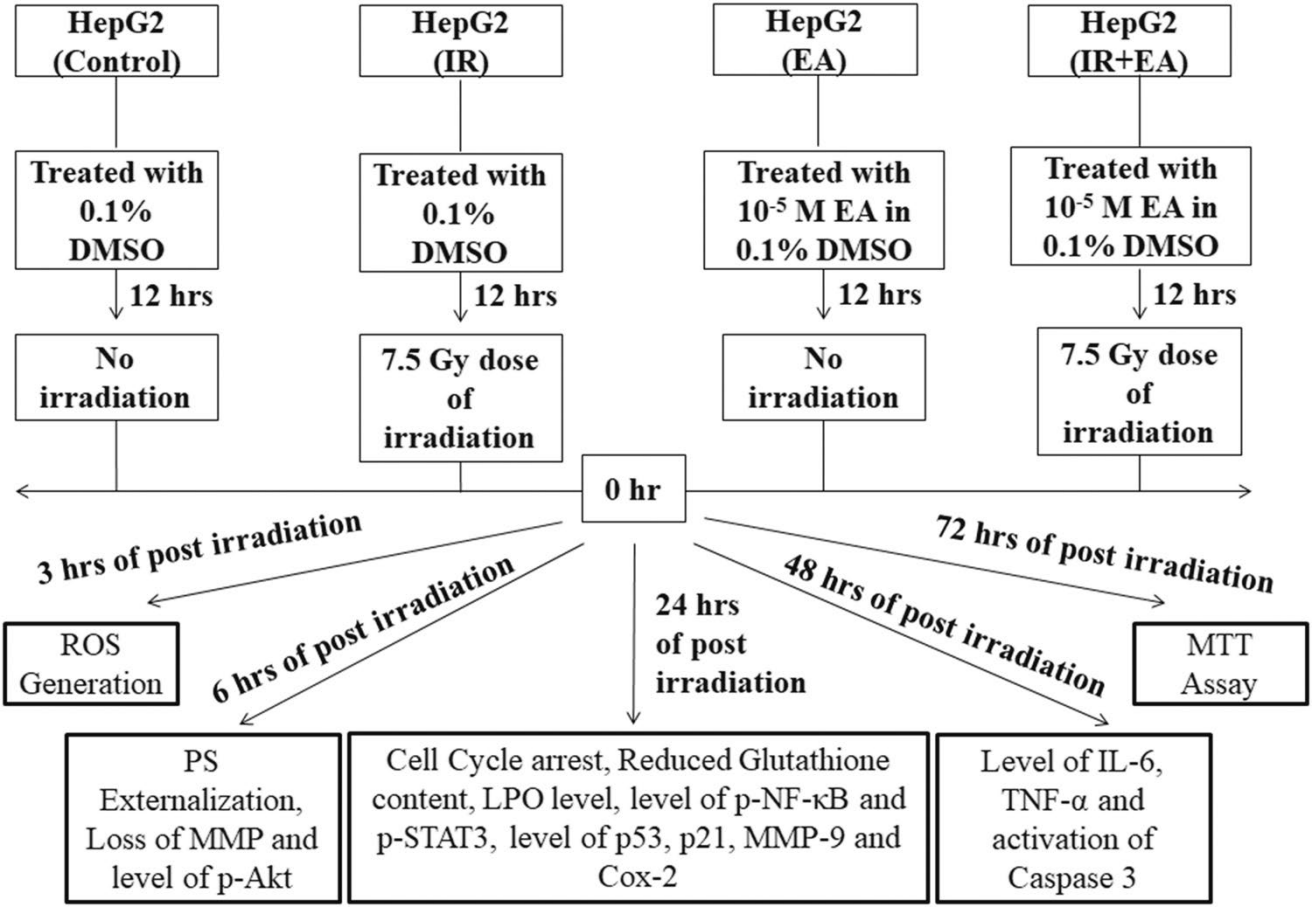
Experimental design. The schematic diagram represented the experimental design of this study. After treatment with EA or vehicle for 12 h the cells were irradiated. The control and only EA treated cells were not exposed to any irradiation. The different experimental parameters were measured at different time points considering the irradiation time point as zero (0) h.

The cells were harvested at different time points. Following harvesting of cells the ROS generation was measured after 3 h of irradiation as it is the immediate early response of radiation exposure. The externalization of PS, MMP and level of p-Akt were measured after 6 h of irradiation. The cell cycle arrest, GSH (reduced glutathione) content, TBARS level, p-NF-κB, p-STAT3, expression of MMP-9, p21, nuclear localization of p53, expression of COX-2 were measured after 24 h of irradiation. The level of active caspase 3, expression of interleukine-6 (IL-6) and tumour necrosis factor- alpha (TNF-α) was measured after 48 h of irradiation (Fig. 1).

### Measurement of intracellular ROS generation

The intracellular ROS was detected by flow cytometry using H_2_DCFDA^27^. H_2_DCFDA, an ROS-sensitive compound diffuses into cells and is hydrolyzed by esterase to form H_2_DCF within cells. H_2_DCF is then oxidized by hydrogen peroxide or low-molecular-weight peroxides to produce the fluorescent compound 2′,7′dichlorofluorescein (DCF)^28^. The trypsinization was performed to collect HepG2 cells after 3 h of radiation treatment to measure intracellular ROS level. Cells were incubated with H_2_DCFDA (3 mM) for 20 min and the fluorescence was measured in FL-1 channel of BD FACS Calibur (Becton Dickinson, NJ, US) flow cytometer equipped with FlowJo software. The assay was repeated for 3 independent times (technical replicate n = 3).

### Determination of reduced glutathione (GSH) activity

To measure the GSH level the cell lysate was treated with 0.1 ml of 25% TCA. The precipitate formed was centrifuged at 3,900 x *g* for 10 mins. The free endogenous sulfhydryl was assayed in mixture of a volume of 1 ml (20 µl of 0.5 mM DTNB prepared in 0.2 M phosphate buffer, with 25 µl of cell supernatant and 955 µl of reaction buffer). Free SH group of reduced glutathione reacts with DTNB formed a yellow complex. The absorbance was read at 412 nm^29^. The assay was performed 3 independent times (n = 3) to calculate the mean ± SEM value.

### Determination of lipid peroxidation

The thiobarbituric acid reactive substance (TBARS) level in the cell lysate was estimated according to the modified protocol of Beuege and Aust^30^. Briefly, the homogenate was mixed with TCA (15%), TBA (0.375%), and HCl (5 N) followed by boiling at 95 °C for 15 mins; Then the mixture was cooled and centrifuged. The absorbance of the supernatant was measured at 535 nm against an appropriate blank. The lipid peroxidation was expressed as the amount of TBARS produced, in nmol/mg protein.

### Measurement of mitochondrial membrane potential changes

The 5,50,6,60-tetrachloro-1,10,3,30-tetraethylbenzimidazolylcarbocyanine iodide (JC-1) stain was used to measure mitochondria membrane potential (ΔΨm) of tumour cells as described previously^31^. The cationic dye, JC-1 normally accumulates in mitochondria to forms JC-1 aggregates (590 nm emission; orange color) in proportion to ΔΨm. When ΔΨm decreases, JC-1 aggregates depart from mitochondria and change to JC-1 monomers (530 nm emission; green color). Therefore, JC-1 was used to detect the occurrence of ΔΨm depolarization in the early stages of apoptosis. 1 × 10^6^ cells were seeded for each experimental group. Briefly, the HepG2 cells were treated with EA (10 µM) for 12 h. After that, cells were exposed to 7.5 Gy dose of irradiation. After 6 h of irradiation, the cells were incubated with JC-1 (2.5 µg/mL) for 20 min at room temperature. The fluorescence of 10,000 cells was measured using a flowcytometer at emission of 525 and 590 nm. The data were analyzed using the software Flowjo 8.0. The assay was performed 4 independent times (n = 4) to calculate the mean ± SEM value.

### Measurement of externalization of phosphatidyl serine (PS) in outer leaflet of biomembrane

The externalization of PS in the outer leaflet of the biomembrane was determined by Annexin-FITC and PI. The cells accepting the colour of FITC represented the early apoptotic population and cells accepting both the colour of FITC and PI represented the late apoptotic population. 1 × 10^6^ cells were seeded for each experimental group. Briefly, after 12 h of EA (10 µM) treatment, cells were exposed to 7.5 Gy dose of irradiation. After 6 h of irradiation, the cells in different experimental groups were incubated with annexin-FITC and PI for 20 min at room temperature. The fluorescence of 10,000 cells was measured using a flowcytometer. The data were analyzed using Flowjo 8.0 software^32^. The assay was performed 3 independent times (n = 3) to calculate the mean ± SEM value.

### Homogenate preparation

For the homogenate preparation we followed the protocol of previous study^33^ with some modification. In brief, HepG2 cells (2 × 10^6^) were suspended in 70 µl of cytosolic extraction buffer (10 mM HEPES, 1.5 mM MgCl_2_, 10 mM KCl and 1 mM dithiothreitol, pH 7.9) containing a protease inhibitor cocktail, and the outer membranes were disrupted by sonication (20 kHz ultrasonic pulse). The supernatant was collected as a cytosolic fraction, by centrifugation at 10,000 x *g* for 20 min at 4 °C. The resultant nuclear pellet was washed to remove cytosolic contaminants and was resuspended in 40 µl of nuclear extraction buffer (20 mM HEPES, 1.5 mM MgCl_2_, 0.42 M NaCl, 0.2 mM EDTA, 1 mM dithiothreitol and 25% (v/v) glycerol, pH 7.9) containing the protease inhibitor cocktail, and sonicated. Finally, the nuclear suspension was centrifuged at 20,000 X *g* for 5 min at 4 °C to collect the supernatant as a nuclear fraction. The tubes were placed in ice during sonication (20 kHz) process and short pulses of 5 seconds were given intermittently for maximum 10 minutes time period. All the processes were done either at 4 °C or in ice^26^. Protein concentration was determined by following the protocol of Lowry *et al*. 1951^34^.

### Western blot assay

For the analysis of protein expression, equal amounts of protein (50 µg) from different cell lysates was loaded in 10% sodium dodecyl sulfate-polyacrylamide gel electrophoresis (SDS-PAGE). The proteins were transferred to activated PVDF membrane after electrophoresis. The membrane was blocked overnight at 4 °C with 5% bovine serum albumin (BSA) solution. Immunoblotting was done as described previously^33^ using monoclonal antibody to p53, p21, Bax, BCL2 MMP-9 and COX-2. β-actin and Histone 3 (H3) antibodies were used as loading control for cytosolic and nuclear extracts respectively. After incubation with primary antibody for 3 h at room temperature, the secondary antibody tagged with alkaline phosphatase was added and incubated for 2 h at room temperature. Protein bands were visualized using an NBT-BCIP solution. The relative protein levels were calculated by normalization to the amount of internal control proteins and were analyzed using GS-700 imaging densitometer and Molecular Analyst software (version 1.5, Bio-Rad Laboratories, Hercules, CA, USA). The western blot was performed for 4 independent times (n = 3) to calculate the mean ± SEM value.

### Immunofluorescence

After 24 h of radiation, cells were fixed with 4% paraformaldehyde, permeabilized with 0.2% Triton X-100 and a blocking solution (2% BSA and 0.1% Triton X-100 in PBS) was used to block nonspecific binding. The fixed cells were then incubated with anti p-STAT3 antibody (at a ratio of 1:250 in the blocking solution), followed by incubation with Rhodamine 1,2,3-labeled anti-mouse secondary antibody (at a ratio of 1:250 in the blocking solution). DAPI was used to stain nuclei. The slides were washed with PBS to remove non specific binding and covered with mounting solution. Finally, the cells were visualized using the fluorescence microscope^35^. Four independent sections (n = 4) were studied to check the nuclear localization of STAT3.

### Cell cycle analysis

2 × 10^6^ cells were seeded for each group. After 24 h of irradiation cells were collected from the different treatment groups and washed twice with cold PBS containing 3% FBS. The cells were then fixed in 70% ethanol overnight at 4 °C. Centrifugation was performed for 5 min at 1,000 rpm (4 °C). The resulting cell pellet was treated with 2 mg/ml RNase A at 37 °C for 20 min and stained with 50 mg/ml propidium iodide (PI) containing 0.1% Triton X-100 and EDTA (0.02 mg/ml). The distribution of cells (10^4^) in different phases of cell cycle was examined using a BD FACSAria III flow cytometer (Becton Dickinson, Franklin Lakes, NJ, USA). The results were analyzed using the FlowJo software^36^. The experiment was repeated for 4 independent times (n = 4).

### Analysis of protein expression by flowcytometry

The cells were harvested after different time points of irradiation and fixed by 4% paraformaldehyde in PBS (pH 7.4). For the permeabilization the cells were incubated with 0.1% Triron X-100 in PBS for 5 min. After washing twice in PBS containing 3% FBS, the permeabilized cells were incubated with primary antibodies (p-Akt, p-NF-kB, active caspase 3 and p-STAT3) for 2 h in ice followed by wash in PBS for two times. The cells were then incubated with either goat anti rabbit or rabbit anti mouse secondary antibody (depending upon the primary Antibody) tagged with FITC fluorescence for 30 mint in ice. The cells were washed with PBS for two times to remove the excess unbound secondary antibody. 10,000 cells for each group were acquired and analyzed by BD FACS calibur equipped with Flow Jo^37^. The expression of different proteins was checked for 4 independent times (n = 4) to calculate mean and SEM.

### Enzyme linked immunosorbant assay

The levels of TNF-α, IL-6 were measured from extracellular medium using a sandwich ELISA Kit (Quantikine M; R & D systems, Minneapolis, MN, USA). The assay was performed as per the detailed instructions of the manufacturer^33^ for 4 independent times.

### Statistical analysis

The values were given as mean ± standard error of the mean (SEM as error). One-way analysis of variance (ANOVA) with Tukey’s post hoc test was done for statistical evaluation of the data and for the determination of level of significance in various groups using the software OriginLab 8.0. In all cases, a value of p < 0.05 was considered as significant. The significance of differences was calculated between the ‘*’ control vs irradiation group and ‘#’ irradiation vs combination (IR+EA) group.

## Results

### Radiation exposure & EA treatment decreased HepG2 cell survivability

**Figure 2:**
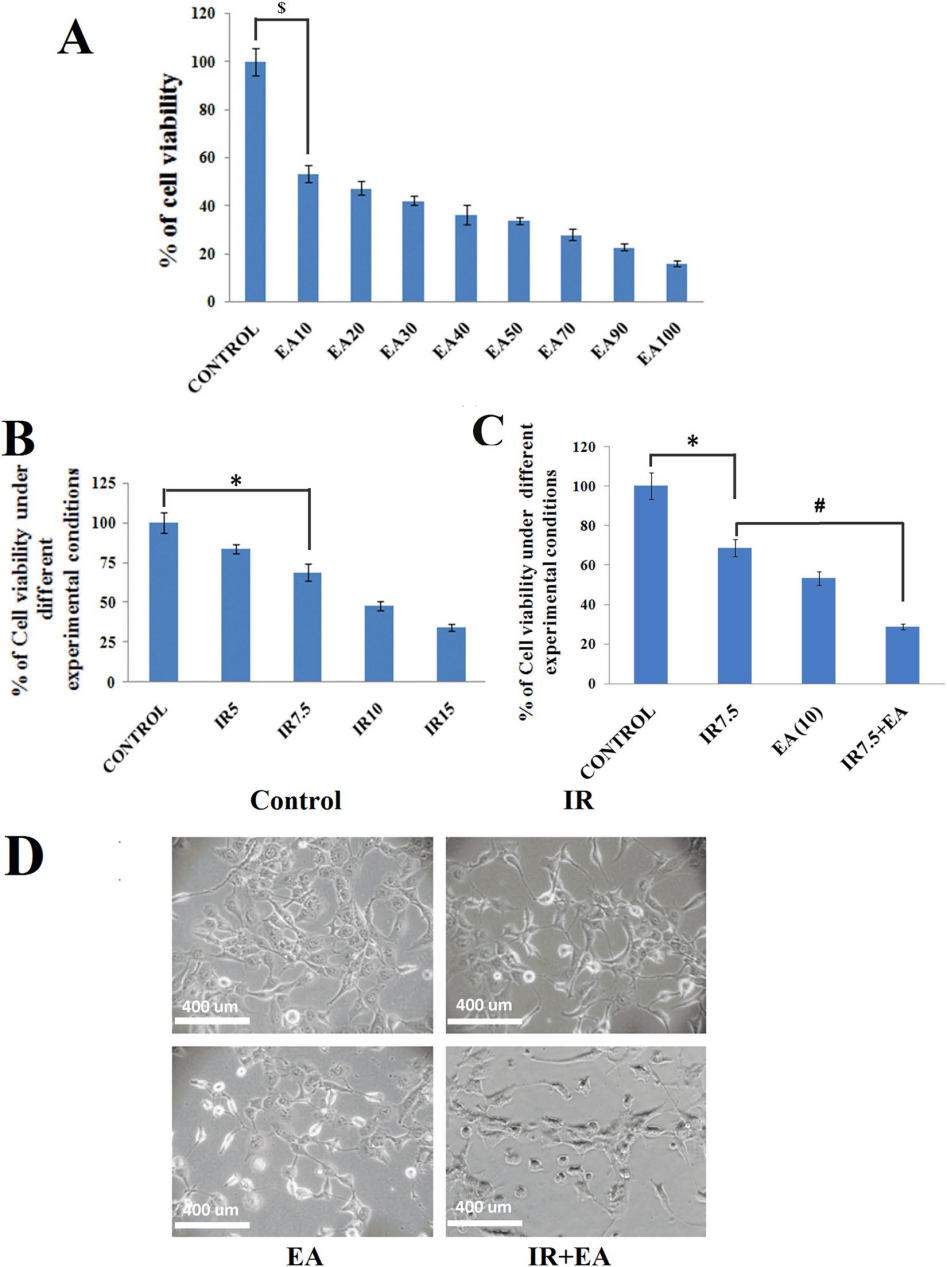
Determination of cell survivability by MTT assay. (**A**) HepG2 cells were treated with increasing concentration of EA for 72 h and cell viability was measured by MTT assay. Y-axis represented the percentage of cell viability and X- axis represented concentrations of EA. (**B**) HepG2 cells were exposed with 5, 7.5, 10 and 15 Gy doses of irradiation and viability was checked after 72 h by MTT assay. The Y-axis represented the cell viability and the X-axis represented the doses of radiation. (**C**) The Bar diagram represented the viability of HepG2 cells after radiation and IR+EA treatment. (**D**) Bright field microscopic images of HepG2 cells after 48 h of EA, IR and IR+EA treatment. Magnification was 400X. Error bars were SEM for n = 3. *p* < 0.05 was considered significant. Statistical comparison was done between control vs. IR designated by ‘*’, control vs EA designated by '$' and IR vs. EA+IR designated by ‘#’ in figure.

The effect of various concentrations of EA on HepG2 cells proliferation was determined by MTT assay. The cells were treated with 10 to 100 µM concentrations of EA and viability was analysed for the measurement of cell proliferation after 72 h of treatment. The viability was significantly (p < 0.05 was considered as significant) decreased to 53.25 ± 3.51% in presence of only 10 µM concentration of EA (Fig. 2A) and therefore, considered as LD_50_ dose of EA for HepG2 cells. The statistical comparison between control and EA (10 µM) was indicated by ‘$’ sign in Fig. 2A. As the lowest dose of EA showed significantly difference in survivability with control group, higher doses also revealed similar results. The proliferation of HepG2 cells was also determined after irradiation. The viability was checked after 72 h following exposure of the cells to different doses of radiation (5 to 15 Gy). The viability was decreased to 34.03 ± 2.36% after 72 h of 15 Gy dose of irradiation. The 7.5 Gy dose of radiation decreased the viability to 68.68 ± 5.39%. 7.5 Gy dose was the not the LD_50_ dose according to MTT data (Fig. 2B). Trypan blue exclusion assay showed reduced growth rate of the cells irradiated with 7.5 Gy^38^. However, cell death was rare at this dose after 72 h. In IR+EA co-treated group the viability was significantly (p < 0.05) decreased to 28.8 ± 1.5% after 72 h of irradiation which indicated more severe inhibition of cell proliferation compared to irradiated group (Fig. 2C). The viability of control cell was considered as 100%. The cellular morphology was severely altered in IR+EA treated group compared to IR and EA treatment alone (Fig. 2D).

### EA in combination with irradiation enhanced intracellular ROS level in HepG2 cells

**Figure 3:**
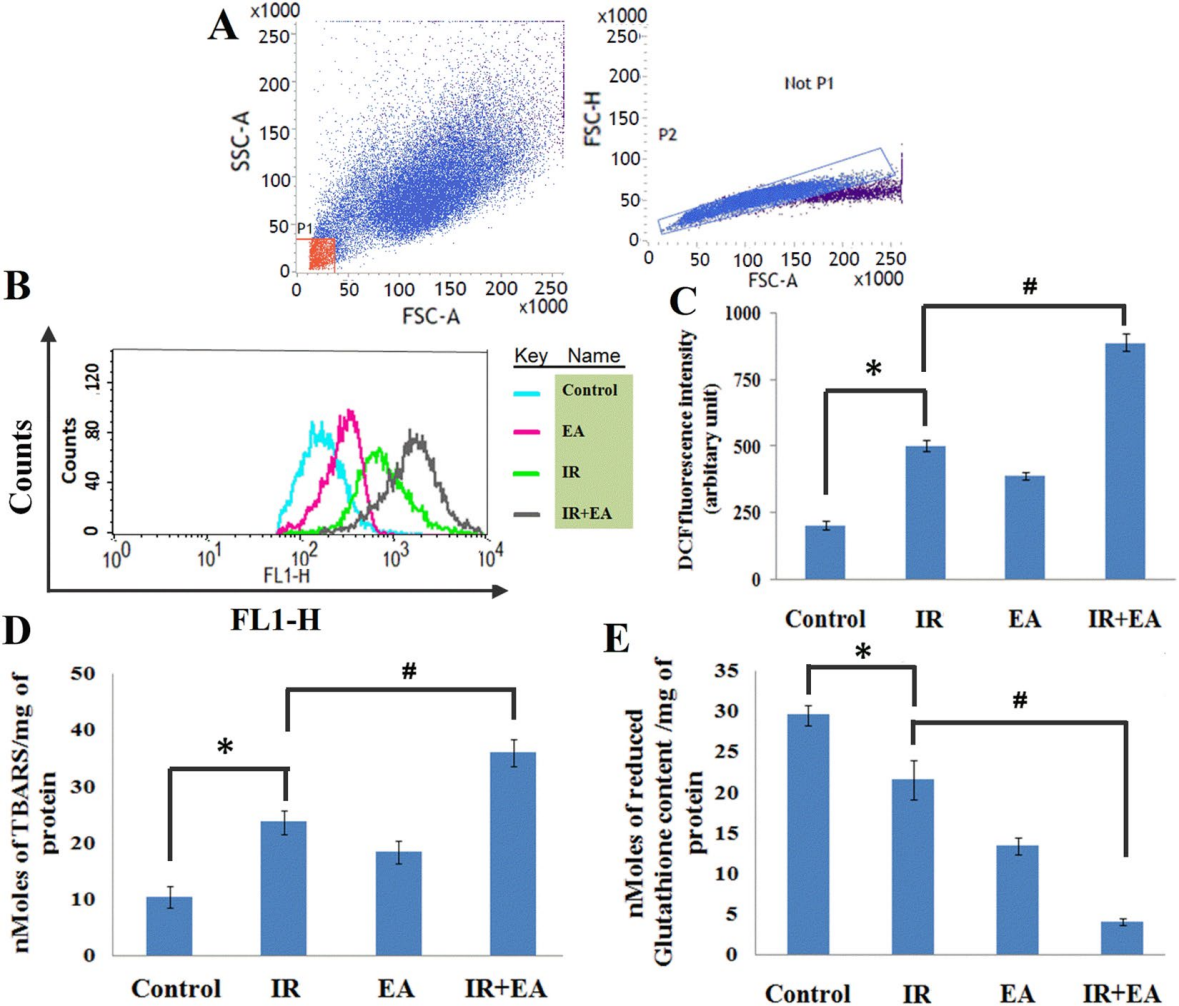
Determination of oxidative stress in HepG2 cells. (**A**) Graph showing separation of cell debris from total cell populations by using FSC-A vs FSC-H plot. (**B**) Overlaid histogram plot of ROS generation in control, IR, EA and IR+EA treated group. The movement of histogram towards right indicated the higher ROS generation. DCF intensity was taken along the X axis, FL1-H (green) channel and count was taken along the Y-axis. Different colours of histogram represented the ROS generation in different experimental groups. (**C**) The Bar diagram represented the mean fluorescence intensity of DCF. IR+EA group showed highest DCF fluorescent intensity among all other groups. (**D**) Bar diagram represented the TBARS level in Control, IR, EA and IR+EA treated groups. Nanomoles of TBARS/ mg of protein had been plotted along the Y-axis and Control, IR, EA and IR+EA were taken along the X-axis. (**E**) Bar diagram represented the reduced glutathione content in control, IR, EA and IR+EA treated groups. Nanomoles of reduced glutathione/mg of protein were plotted along the Y-axis and Control, IR, EA and IR+EA were taken along the X-axis. Error bars were SEM for n = 3. *p* < 0.05 was considered significant. Statistical comparison was done between control vs. IR designated by ‘*’, and IR vs. EA+IR designated by ‘#’ in figure.

After optimization of radiation and EA dose for the sensitization of HepG2 cells to irradiation, the ROS generation was measured by flowcytometry using H_2_DCFDA in irradiated (IR), EA treated and combined (IR+EA) group (Fig. 3B). Treatment of these tumour cells with EA (389.4 ± 14.69 DCF fluorescence intensity in arbitrary unit) showed a significant (p < 0.05 was considered as significant) increase in ROS generation with respect to control (204.75 ± 15.07) group (Fig. 3C). Moreover, combined treatment showed additive effect in the intracellular ROS generation. A significant increase in ROS production by 4.34 fold compared to control was found after treatment of cells with 10 μM of EA and a single dose of 7.5 Gy (Fig. 3C). The formation of ROS in IR+EA group was substantially high (890.19 ± 29.04) (p < 0.05 was considered as significant) compared to the cells treated with either radiation (503.5 ± 18.7) or EA (389.4 ± 14.69). In Fig. 3B the overlaid histogram showed the ROS generation in different experimental group and the bar diagram (Fig. 3C) showed the DCF fluorescence intensity of different groups.

### EA increased TBARS level in HepG2 cells after irradiation

The TBARS level was checked after 24 h of irradiation. The cells were incubated with EA for 12 h prior to irradiation. The irradiated cells showed high TBARS level compared to control group. EA treatment alone also increased ROS mediated lipid peroxidation in HepG2 cells. EA together with radiation (IR+EA) increased TBARS level significantly (p < 0.05) compared to the only irradiated group (Fig. 3D). This implied that co-treatment increased the oxidative stress situation in HepG2 cells which may lead to apoptosis of HepG2 cells.

### EA decreased the reduced glutathione content in HepG2 cells after irradiation

The GSH level was checked after 24 h of irradiation using Elman’s reagent by colourimetric assay. The EA treated cells showed lower GSH level compared to control group. The combination treatment (IR+EA) showed significantly (p < 0.05) low level of GSH compared to the only irradiated group (Fig. 3E). This further supported that co-treatment with IR and EA induced more oxidative stress situation HepG2 cells compared to irradiation alone.

### Combination of EA and radiation decreased the p-Akt level and increased apoptotic population in HepG2

**Figure 4:**
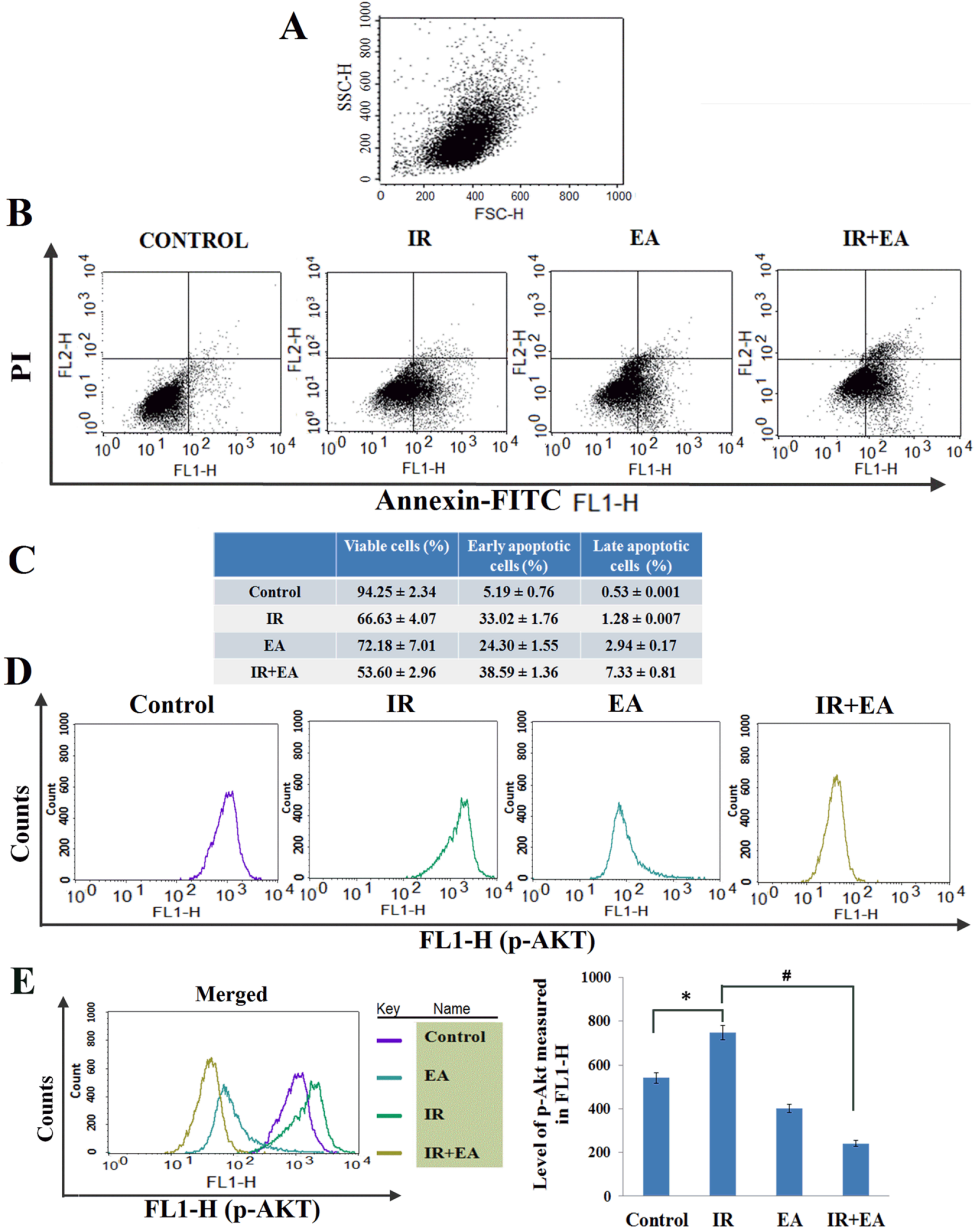
Determination of PS externalization and p-Akt level: (**A**–**C** externalization of PS), (**D**,**E** p-Akt). (**A**) Graph represented the cell population on the basis of size (FSC-H) and granularity (SSC-H). (**B**) HepG2 cells of control, IR, EA and IR+EA treated groups were stained by Annexin-FITC and PI. The Q1 quadrant represented the viable cell populations which was the maximum in Control group. Q2 quadrant represented the early apoptotic cell populations with FITC stain. The Q3 quadrant represented the late apoptotic cell population (dual stain positive cells). Intensity of FITC in FL1-H (FITC) channel was taken along the X-axis and FL2-H channel (PI) was taken along the Y-axis (**C**) Table represented the viable, early apoptotic and late apoptotic cell populations in control, IR, EA and IR+EA treated groups. Error bars were SEM for n=3. p<0.05 was considered significant. (**D**) The histogram plot of control, IR, EA and IR+EA treated groups represented the p-Akt level. The right shift of histogram indicated higher p-Akt level. Along the X-axis intensity of FITC in FL1-H (FITC) channel and along the Y-axis counts were taken (**E**) Different colours of histogram in overlaid plot indicated the p-Akt level in different groups. Error bars were SEM for n = 4. *p* < 0.05 was considered significant. Statistical comparison was done between control vs. IR designated by ‘*’, and IR vs. EA+IR designated by ‘#’ in figure.

The expression of p-Akt and the externalization of phosphatidyl serine (PS) in the outer leaflet of the bio-membrane were examined by flowcytometry. The combination treatment showed higher percentage (p < 0.05 was considered as significant) of early apoptotic (38.59 ± 1.36%) and late apoptotic cells (7.33 ± 0.81%) compared to only irradiated group (33.02 ± 1.72% of early apoptotic cells and 1.28 ± 0.007 of late apoptotic cells) (Fig. 4B,C). The p-Akt level was measured after 6 h of irradiation. The histogram plot of each group represented the level of p-Akt (Fig. 4D). p-Akt level was decreased by only EA treatment but increased (775 ± 25.02) significantly (p < 0.05) by 1.28 fold after irradiation (Fig. 4E). EA in combination with radiation (295 ± 14.34) showed a massive decrease (2.31 fold) in the phosphorylation of this crucial survival marker in this tumour cells. Thus, EA along with irradiation significantly decreased p-Akt level which was up-regulated by radiation alone.

### Alteration in mitochondrial membrane potential (ΔΨm) by radiation and combination treatment

**Figure 5:**
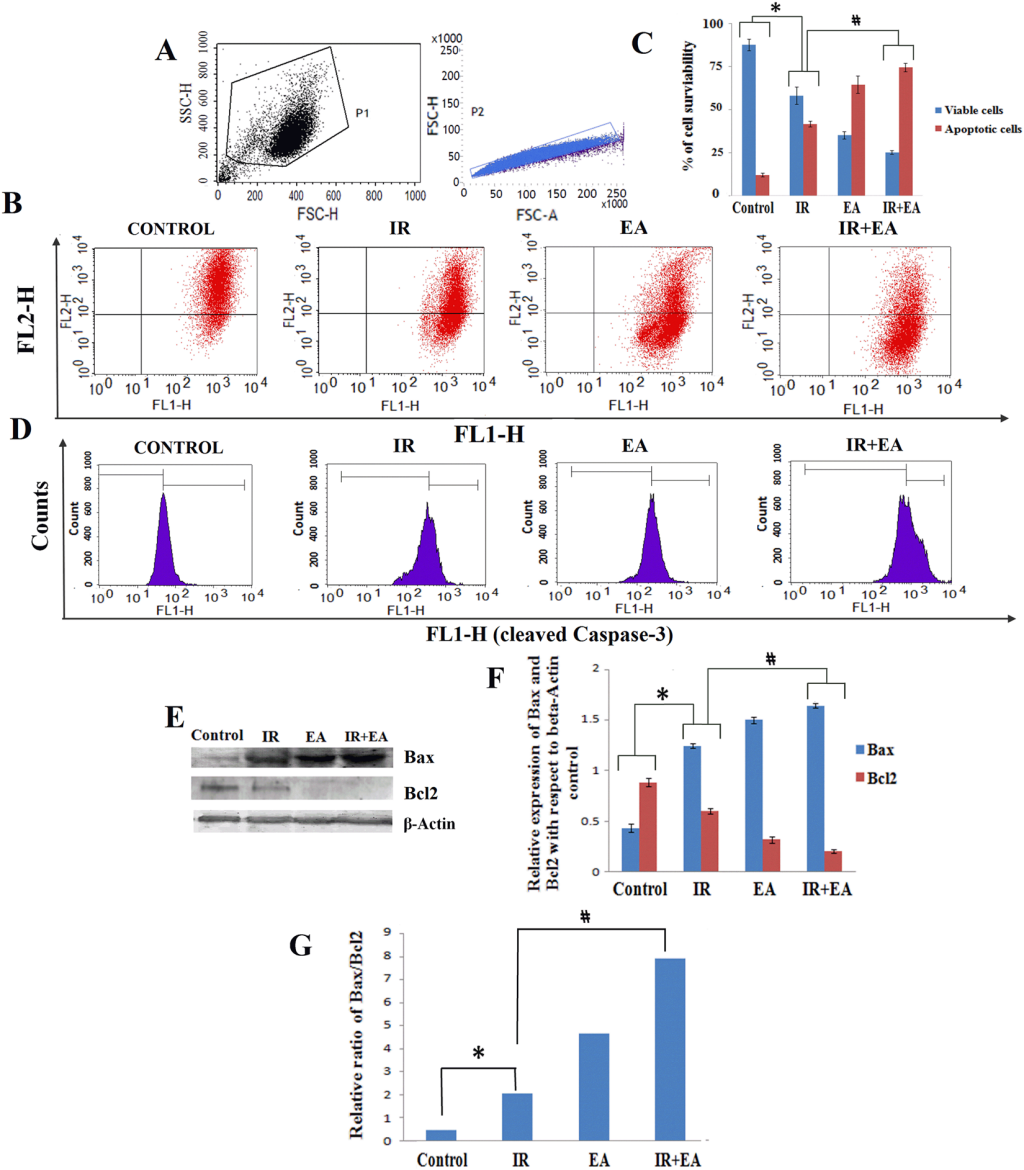
Combination treatment induced mitochondrial apoptosis pathway. (**A**) Graph showing separation of cell debris from total cell populations by using FSC-H vs FSC-A plot. (**B**) The cells present in Q2 quadrant represented the cells with loss of MMP and apoptotic cells. The cells present in Q3 quadrant represented viable cells with no loss of MMP. As the cells loose MMP, JC1 dye showed green colour instead of red and cells came into lower Q2 quadrant. Along the X-axis intensity of FITC in FL1-H (FITC) channel and along the Y-axis FL2-H channel (Red) was taken **C**. The bar diagram represented the percentage of viable and apoptotic cell populations. The red bars stand for apoptotic population and blue bars stand for viable population. (**D**) The histogram plot represented the level of active caspase 3 in control, IR, EA and IR+EA treated groups. The right shift of histogram indicated higher level of active caspase 3. Along the X-axis intensity of FITC in FL1-H (FITC) channel and along the Y-axis count was taken (**E**). The diagram showed immunoblot images of Bax, BCL2 and beta-Actin. (**F**) Bar Diagram represented the Bax and BCL2 expression level in control, IR, EA and IR+EA treated groups. The blue bars stand for Bax expression and red bars stand for BCL2 expression. (**G**) Bar diagram represented the Bax/Bcl2 ratio in different experimental groups. Error bars were SEM for n = 4. *p* < 0.05 was considered significant. Statistical comparison was done between control vs. IR designated by ‘*’, and IR vs. EA+IR designated by ‘#’ in figure.

JC-1 is a cationic dye accumulates in mitochondria to form JC-1 aggregates (590 nm emission; orange colour) in proportion to ΔΨm. When ΔΨm decreases, JC-1 aggregates depart from mitochondria and change to JC-1 monomers (530 nm emission; green color). The cells lost their potential after treatments were designated as apoptotic cells and rest designated as viable cells. Radiation alone showed 41.75 ± 1.66% cell population in Q2 quadrant represented the loss of membrane potential (Fig. 5B). Moreover, EA plus radiation treatment significantly (p < 0.05) increased the population in Q2 quadrant to 74.66 ± 2.37% (Fig. 5B). The bar diagram in Fig. 5C represented the percentage of viable and apoptotic cells with loss of MMP. EA treatment alone showed 64.65 ± 4.98% cells with loss of membrane potential. Therefore, loss of membrane potential after combined treatment was more in HepG2 cells compared to EA or IR treatment alone.

### Combination treatment increased the Bax/Bcl2 ratio and active caspase 3 level

Bax and BCL2 expression were studied by Immunoblot. The combination treatment showed significantly high level Bax expression (Fig. 5E) with an expected decrease in BCL2 expression (Fig. 5E). Thus, the Bax/BCL2 ratio had significantly (p < 0.05) increased in combination group by 3.8 fold compared to the irradiation alone (Fig. 5G). The densitometric analysis of immunoblot revealed that combination treatment increased the Bax expression in HepG2 cells by 1.31 fold compared to IR group (Fig. 5F). Similarly, the BCL2 level was compromised significantly (p < 0.05) due to combination treatment by 2.91 fold (Fig. 5F). The level of active caspase 3 followed the similar trend. The active caspase 3 level was increased by combination treatment and which was 1.5 fold higher compared to radiation treatment alone (Fig. 5D).

### Alteration of cell cycle progression of HepG2 cells by radiation and EA treatment

**Figure 6:**
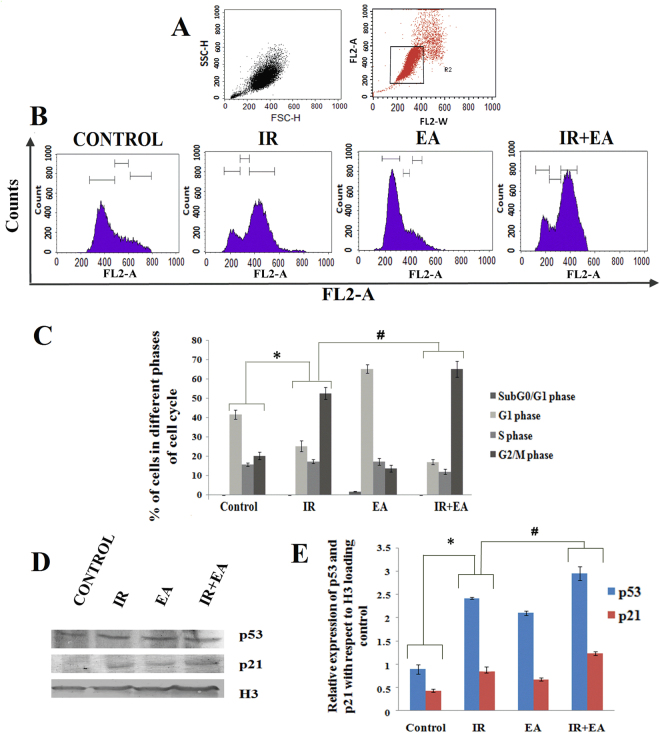
Determination of cell cycle progression, p53 and p21 expression. (**A**) Graph represented the cell population on the basis of size (FSC-H) and granularity (SSC-H). The selected population was plotted in FL2-A vs FL2-W plot to discriminate singlet and doublet population. Then singlet population was selected and plotted in count vs FL2-Area (Red) graph (linear scale) (**B**). Graphs represented the distribution of cells in different phases of cell cycle. The 1^st^ peak in all graphs represented the G1 population and 2^nd^ peak represented the G2/M population. The valley between these two peaks represented the S phase population. (**C**) The bar diagram represented the % of cells in different phases of cell cycle. (**D**) The Immunoblot images of the p53, p21 and H3 in control, IR, EA and IR+EA treated groups. (**E**) The bar diagram represented the expression of p21 and nuclear translocation level of p53. The blue bars represented the p53 protein and red bars represented p21 protein. Error bars were SEM for n = 4. *p* < 0.05 was considered significant. Statistical comparison was done between control vs. IR designated by ‘*’, and IR vs. EA+IR designated by ‘#’ in figure.

The cells were incubated with EA for 12 h prior to irradiation and the cell cycle progression was analysed after 24 h of 7.5 Gy dose of irradiation. The panel 6 A represented the cell population and doublet discrimination plot. The singlet cell population was chosen to examine the cell cycle progression. The control group showed 41.64 ± 2.35% cell population in G1 phase and 20.29 ± 1.4% population in G2/M phase. Irradiated group showed a significantly high cell population (56.36 ± 1.39%) in G2/M phase represented the G2/M phase arrest. But, IR+EA showed 65.32 ± 4.07% cell population at G2/M phase (Fig. 6B,C), indicated combination treatment showed higher percentage of G2/M phase population compared to IR alone (p < 0.05 was considered as significant). Only EA treated group showed 65.34 ± 2.3% population in G1 phase and 3.10 ± 0.052% sub G_0_/G1 cell population which was absent in all other groups (Fig. 6B,C).

### Combination and EA treatment increased the p53 and p21 levels in HepG2 cells

Irradiation was responsible for DNA damage which stabilized p53 and the later subsequently translocated into nucleus. EA and IR+EA treated groups showed increased nuclear translocation of p53 among all other groups (Fig. 6D). The combination group showed 1.2 fold higher p53 level (p < 0.05) in the nucleus compared to the irradiated group as obtained from densitometric analysis (Fig. 6E). EA treated group showed approximately similar level of p53 like irradiated group. The Immunoblot data also showed the combination treatment increased the p21 expression (Fig. 6D). IR+EA treated group showed 6.35 fold higher p21 level (p < 0.05) compared to radiation alone (Fig. 6E). Thus, EA treatment prior to irradiation significantly up regulated p53 and p21 levels in tumour cells.

### EA along with radiation decreased p-NF-κB level in tumour cells

**Figure 7:**
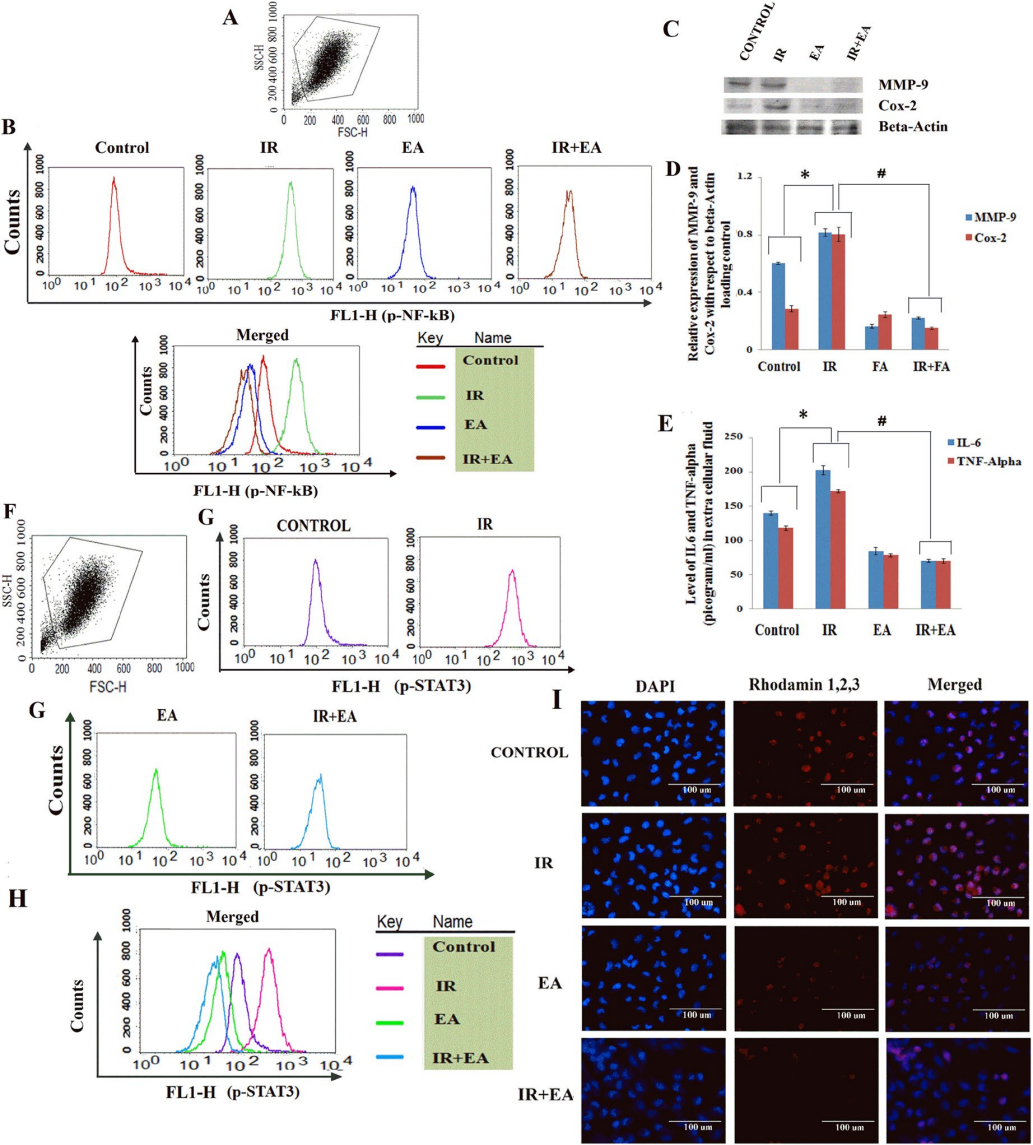
Combination treatment decreased inflammation and angiogenesis markers expression. (**A**) Graph represented the cell population on the basis of size (FSC-H) and granularity (SSC-H). (**B**) The histogram plots represented the p-NF-κB level in control, IR, EA and IR+EA groups. The shift of histogram towards right indicated higher p-NF-κB level. Along the X-axis intensity of FITC in FL1-H (green) channel and along the Y-axis count was taken. The merged image showed the differences in histogram of different experimental groups. (**C**) Immunoblot image of MMP-9 and COX-2 with beta-Actin loading control. (**D**) The densitometry plot of MMP-9 and COX-2. Blue bars stand for MMP-9 and red bars stand for COX-2 protein. (**E**) Bar diagram represented the level of IL-6 and TNF-α in extra cellular fluid after 48 h of irradiation. The blue bars stand for IL-6 and red bars stand for TNF-α. (**F**) Graph showing removal of cell debris from total cell populations by using FSC-H vs SSC-H plot. (**G**) The histogram plots represented the p-STAT3 level in control, IR, EA and IR+EA treated groups. The shift of histogram towards right indicated higher p-STAT3 level. Along the X-axis intensity of FITC in FL1-H (green) channel and along the Y-axis count was taken. (**H**) The different colours of histogram in merged image represented differences in the histograms of different experimental groups. (**I**) Immunocytochemistry image of control, IR, EA and IR+EA treated groups. The nuclei were stained by DAPI which appeared blue and p-STAT3 was stained by Rhodamine 1,2,3 which appeared red. The merged images showed the infiltration of red colour into the blue region which indicated the nuclear translocation of STAT3. Error bars were SEM for n = 4. *p* < 0.05 was considered significant. Statistical comparison was done between control vs. IR designated by ‘*’, and IR vs. EA+IR designated by ‘#’ in figure.

NF-κB is a robust transcription factor playing key role in cell survival and inflammation. Activation of this transcription factor is regulated by upstream signaling molecules that are activated by external stimuli. Upon activation NF-κB gets phosphorylated and translocated to nucleus. Thus we checked the level of p-NF-κB from fixed HepG2 cells by flowcytometry. The control group showed a basal level of p-NF-κB (539 ± 12.62) which was significantly (p < 0.05) up-regulated after 24 h of radiation (756.69 ± 17.95) (Fig. 7B). The activated NF-κB may increase tumour cell survival and onset of inflammatory pathway. The combination treatment (391.31 ± 11.05) and EA treatment alone altered the p-NF-κB level in HepG2 cells which was significantly (p < 0.05) lower compared to the control and irradiated group.

### EA along with radiation decreased inflammation and MMP-9 expression in tumour cells

The expression of IL-6 and TNF-α was measured by sandwich ELISA (Fig. 7E) and expression of COX-2 and MMP-9 was measured by immunoblot (Fig. 7C). Irradiation caused higher expression of both IL-6 and TNF-α in tumour cells. The Irradiated group showed 2.88 fold higher (p < 0.05) expression of IL-6 compared to the IR+EA treated group (Fig. 7E) whereas, TNF-α level was 2.44 fold higher (p < 0.05) in irradiated group compared to co-treated (IR+EA) group (Fig. 7E). The expression of COX-2 inflammatory and MMP-9 angiogenesis markers were increased significantly (p < 0.05) in irradiated group by 3.65 (Fig. 7C,D) and 5.27 fold (Fig. 7C,D) respectively compared to co-treated group. Therefore, co-treatment reduced the COX-2 mediated survival response and also angiogenesis pathway activation.

### Radiation and EA combination treatment decreased p-STAT3 level in tumour cells

p-STAT3 level was measured from tumour cells after 24 h of irradiation using flowcytometry. STAT3 is another survival modulating factor known to involve in inflammatory pathway. After 24 h of irradiation the p-STAT3 level (735.8 ± 27.59) was significantly (p < 0.05) increased in irradiated group compared to the control group. As shown in Fig. 7G,H, the level of p-STAT3 was significantly (p < 0.05) compromised by IR+EA treatment (272.11 ± 14.42) compared to radiation alone. The histogram plot (Fig. 7G,H) and intensity value also revealed that combination treatment showed a notable decrease in p-STAT3 level compared to the EA alone (384.02 ± 13.36) and thus, depicted EA along with radiation was more effective to tumour cells than radiation or EA alone.

The nuclear localization of p-STAT3 was studied by immunocytochemistry which corroborated the data obtained by flowcytometry. In flowcytometry we checked only the activated STAT3 level but the nuclear translocation of STAT3 was confirmed by microscopic data. Irradiation caused enhancement of p-STAT3 nuclear translocation after 24 h which was clearly visible in the merged image of DAPI and Rhodamin1,2,3 stained HepG2 cells (Fig. 7I). EA and combination treatment significantly (p < 0.05) decreased this translocation process however the decrease was more robust in radiation plus EA treatment than EA alone. The number of STAT3 positive nuclei was significantly high in IR group compared to IR+EA and EA treatment group.

## Discussion

Our study aimed to investigate the effect of EA on cell death and proliferation of liver cancer cells and to determine the mechanism through which EA affects cell survival. We used the highly differentiated HepG2, human hepatocellular carcinoma cell line as a model. We found that EA in combination with radiation: (a) stimulated ROS generation and inhibited proliferation of HepG2 cells by decreasing p-Akt level; (b) activated the mitochondrial death pathway associated with loss of ΔΨm, caspase-3 activation, higher Bax/BCL2 ratio.; (c) decreased p-Akt, p-NF-κB and p-STAT3 levels in cancer cells; (d) activated p53 and p21; (e) inhibited the onset of secondary survival pathway by decreasing IL-6, COX-2 expression and (f) prevented angiogenesis marker MMP-9 expression.

Polyphenols are known to enhance the effects of radiation^21,25,39^. Tumour cells showed response to the treatment of either radiation or EA, which is implicated in its potential of increasing intracellular ROS generation, but more pronounced response was seen in cancer cells treated with the combination of radiation and EA. It is acknowledged that for many but not all signals, the balance between the competing activities of the pro-proliferative/anti-apoptotic and pro-apoptotic proteins determine the fate of cell^40^. We found that HepG2 cells in culture condition showed increased cytotoxic response to radiation plus EA treatment as compared to the untreated cells and those treated with either radiation or EA. The increased pro-oxidant activity of radiation plus EA group might be the cause of increased cytotoxicity in tumour cells. Earlier report showed that flavonoids like that of EA and silibinin are known to exhibit the pro-oxidant activity and act as an inducer of intracellular oxidative stress in tumour cells which also induces the formation of reactive oxygen species *in vitro* and *in vivo* conditions^41^. There was a decrease observed in the antioxidant enzymes like superoxide dismutase, catalase, and glutathione reductase in tumour cells *in vivo* ^41^. Tanaka *et al*. showed that EA exhibited cancer inhibitory properties. Using gamma-glutamyl transpeptidase reaction, neoplasms and liver-altered foci were identified. Interestingly, the altered foci number was lower at all time points in the group receiving the EA and FAA treatment. The frequency of hepatocellular neoplasms (30%) was found to be reduced^42^. Here we have also noticed the cell mortality was significantly greater in cells treated with EA prior to radiation. A prominent decrease of reduced glutathione content and increase TBARS level observed in cells treated with radiation plus EA. An increase in TBARS levels was observed in cells treated with radiation and EA alone. The decline in GSH levels suggested the decrease in antioxidant capacity of the tumour cells, which predicts the inefficiency of these antioxidant enzymes to suppress the ROS generated by stress in those cells. In normal healthy cells, enzymatic and non-enzymatic antioxidants serve to balance the intracellular production of ROS and its neutralization, thereby delaying or inhibiting the destructive oxidation. The potential for oxidative damage is greatly increased if the antioxidant capacity is insufficiently expressed^43^. Moreover, increased ROS production in IR+EA group experienced additive stress along with the compromised antioxidant capacity in HepG2 cells. Report revealed cell malignancy or transformation is often accompanied by a decrease in activity of antioxidant enzymes (SOD, catalase, GSH-Px, GR), which increased the cell sensitivity to pro-oxidant compounds^44^. The susceptibility of tumour cells to radiation or drug is associated with decreased level of antioxidants^45–49^.

A decrease in mitochondrial potential is known to be associated with cell death either by apoptosis or by necrosis. The HepG2 cells treated with radiation plus EA showed drop in transmembrane mitochondrial potential suggesting the probability of involvement of mitochondrial permeability changes in ROS induced damage in tumour cells. Alteration of membrane potential caused leakage of cytochrome C which is the initiator of intrinsic apoptotic pathway. Radiation plus EA, at a concentration of 10^−5^ M induced G2/M arrest within 48 h, inhibited overall cell growth and induced apoptosis in HepG2 cells after 72 h of treatment. HepG2 cells were found to be sensitive to EA and showed G1 arrest within 24 to 48 h. Flow cytometry analysis revealed the arrest of cells at the G1 phase after 36 h (Fig 1) of EA (10^−5^ M) tratment, whereas the radiation alone and combination treatment showed G2/M arrest of cells after 24 h of irradiation. However, to find out the mechanism of action of EA in inducing cell cycle arrest, we have checked the expression of p53 and p21 in the nuclear extract of HepG2 cells and the maximum expression of p53 and p21 were found in radiation plus EA combination group. It was found that the p53 protein which is a pro- apoptotic protein was seen to be upregulated in HepG2 cells treated with both EA and radiation compared to cells treated with either EA or radiation. HepG2 cells also showed elevated caspase 3 activity after 48 h (Fig 1 and Fig 5)when treated with EA and radiation. Previous report showed that the requirement of p53-mediated transcriptional activation of Bax^50,51^. Similar observation was also found in our study. Overexpression of Bax and higher level of p53 were observed in co-treated group indicated that p53 augmented Bax expression. This increased the Bax/BCL2 ratio. Therefore, more Bax was available to form pores in the mitochondrial membrane and caused the leakage of cytochrome C. Cytochrome C now in turn activated the intrinsic apoptotic pathway which was characterized higher level of active caspase 3 in combination group compared to only IR or EA group.

**Figure 8:**
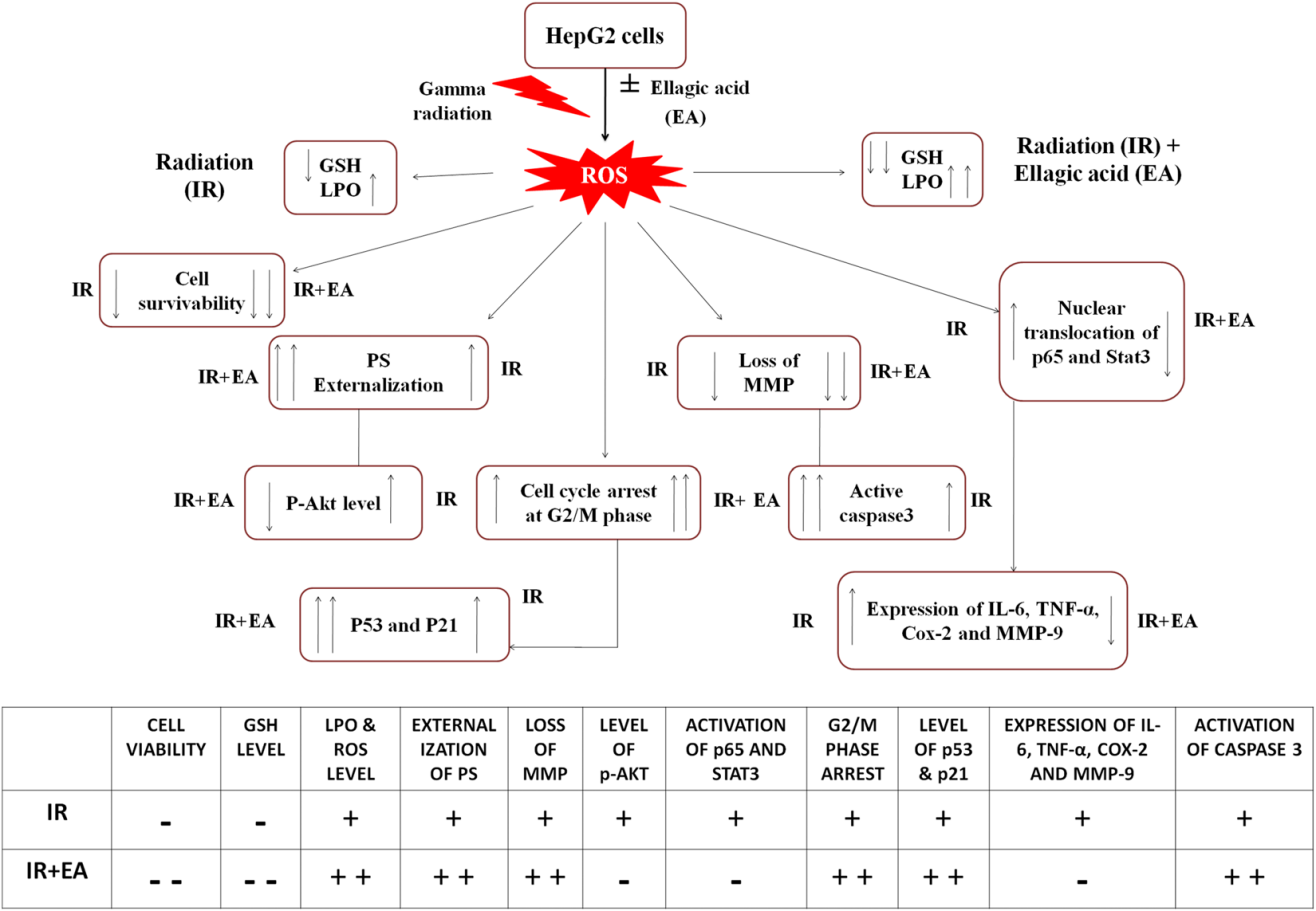
Schematic diagram of the hypothesis. The diagram represented the hypothetical pathways involved in radio-sensitization of HepG2 cells. The double arrow indicated more increase or decrease of a specific marker under IR+EA treated condition. The table in the lower panel elaborately stated the changes of different markers in either irradiated or in irradiation plus EA treated condition. The symbol plus (+) indicated the increased expression or activation of particular markers and negative symbol (−) indicated the decreased expression or activation of particular markers. The symbol double plus (++) in the table indicated more increase or activation of a particular marker compared to single plus (+) symbol. The double negative (−) symbol indicated more decrease or less activation of a particular marker than single negative (−) symbol.

The modulation in the activation of survival markers by EA+IR is not studied yet. Therefore, we explored to find out the impact of EA and co-treatment on cell survival pathway. What we found that irradiation caused NF-κB mediated IL-6 and COX-2 over expression in HepG2 cells. NF-κB is well documented as an important survival marker in cancer cells. Interestingly, IL-6 is known to activate STAT3 which in turn interacts with NF-κB to form ReLA survival complex that gives the radio resistant property to cancer cells^52^. NF-κB mediated TNF-α over expression can augment the further NF-κB activation and thus inducing the activation of survival pathway. The transcription factor STAT3 (signal transducer and activator of transcription) plays a critical role in signal transduction and activation of transcription. Many cytokines including interleukins, growth hormones, epidermal growth factor, platelet-derived factor and interferon function through the STAT signaling pathway^53^. Studies have shown that persistent activation of STAT3 is an important reason of tumor proliferation and metastasis^54^. Combination treatment down regulated NF-κB, IL-6, COX-2 and TNF-α axis that may inhibit ReLA survival complex formation and thus, inhibiting the cell survival pathway that was up-regulated by irradiation alone. The level of p-Akt was also increased after 6 h of irradiation. Eskandari *et al*. in his article showed that EA treatment decreased the p-Akt level in PC3 cells^38^. We observed that both EA and IR+EA treatment decreased the p-Akt level and therefore, suppressed the survival signaling pathway activation. Irradiation caused upregulation in NF-κB mediated MMP-9 expression in HepG2 cell^55^. Similar result was obtained in our study. But we were curious about the fact that whether this combination treatment would able to suppress the MMP-9 expression. The result of immunoblot showed the down regulation in MMP-9 expression by combination indicated the inhibition of angiogenesis pathway activation. Therefore, we were able show how EA in combination with radiation able to create an imbalance between cell survival and apoptosis pathway and shifted the cancer cells towards apoptosis by upregulating p53-p21-Bax-active caspase 3 axis, simultaneously by downregulating NF-κB-COX-2-BCL2-IL6-STAT3 axis (Fig. 8). Therefore, the current article is the first to show the modulation of cell survival and apoptotic pathways together in HepG2 cell by EA, IR and by their combination treatment with detailed mechanistic approach.

The entire strategy of this current study was to develop a unique way to minimize the dose of radiation for killing HepG2 cells with the help of pro-oxidant effects of EA. The HepG2 cells were chosen as hepatocellular carcinoma cells since these are one of the most radio-resistant ones. Perhaps, this is the first report with genuine scientific rationale of radiation sensitization of HepG2 cell by EA, along with evidence based results. The doses were tuned by combining the gamma radiation and EA on HepG2 cells. A reduced dose of 7.5 Gy with EA was capable of showing the effect of 15 Gy of gamma radiation alone (Fig. 2B,C). We documented the hypothesis and proof of scientific concept with most contemporary cellular mechanisms. This strategy was fruitful, as the combined dose prevented not only the survival, but also it enhanced the cell cycle arrest and apoptosis of HepG2. Thus, we are confident that this unique endeavour of combination will remain as a positive footmark in cancer research. In future, *in vivo* studies and clinical trials with this combination will warrant the effectiveness of the current approach.
